# Exposure Definition in Case–Control Studies of Cervical Cancer Screening: A Systematic Literature Review

**DOI:** 10.1158/1055-9965.EPI-21-0376

**Published:** 2021-09-14

**Authors:** Alejandra Castanon, Aruna Kamineni, K. Miriam Elfström, Anita W.W. Lim, Peter Sasieni

**Affiliations:** 1 King's College London, Faculty of Life Sciences & Medicine, School of Cancer & Pharmaceutical Sciences, Cancer Prevention Group, Innovation Hub, Guys Cancer Centre, Guys Hospital, Great Maze Pond, London, United Kingdom.; 2 Kaiser Permanente Washington Health Research Institute, Seattle, Washington; 3 Institutionen för Laboratoriemedicin, Department of Laboratory Medicine, Karolinska Institutet, Stockholm, Sweden.

## Abstract

The first step in evaluating the effectiveness of cervical screening is defining exposure to screening. Our aim was to describe the spectrum of screening exposure definitions used in studies of the effectiveness of cervical screening. This systematic review included case-control studies in a population-based screening setting. Outcome was incidence of cervical cancer. Three electronic databases were searched from January 1, 2012 to December 6, 2018. Articles prior to 2012 were identified from a previous review. The qualitative synthesis focused on describing screening exposure definitions reported in the literature and the methodologic differences that could have an impact on the association between screening and cervical cancer. Forty-one case–control studies were included. Six screening exposure definitions were identified. Cervical cancer risk on average decreased by 66% when screening exposure was defined as ever tested, by 77% by time since last negative test, and by 79% after two or more previous tests. Methodologic differences included composition of the reference group and whether diagnostic and/or symptomatic tests were excluded from the analysis. Consensus guidelines to standardize exposure definitions are needed to ensure evaluations of cervical cancer screening can accurately measure the impact of transitioning from cytology to human papillomavirus–based screening and to allow comparisons between programs.

## Introduction

Cervical cancer screening has the potential to detect both asymptomatic cancers and precancerous lesions, enabling the reduction of cervical cancer mortality and incidence. The case–control study design is efficient when the outcome is rare and/or primary data collection is needed, providing a feasible approach to quantify the benefit of screening in reducing cervical cancer incidence at a population level. Hence this study design is often used for evaluations or audits of cancer screening programmes. Many case–control studies of cervical screening rely on medical records rather than questionnaires to ascertain screening exposure but have characterized screening exposure in different ways. This variation in screening exposure definition combined with other methodologic considerations may lead to different estimates of the benefit of cervical cancer screening in these case–control studies.

The detectable preclinical phase (DPP; ref. 1) refers to the period beginning at the time when a cancerous or precancerous lesion is detectable by screening and ending with the onset of clinical signs or symptoms of invasive cancer (Supplementary Fig. S1). Ideally case–control studies evaluating the association between screening and cervical cancer incidence should aim to compare screening histories of cases and controls during the subperiod of the DPP in which only precancerous lesions are present. It is only during the precancerous phase that screening can lead to the detection and treatment of lesions to prevent cancer (2). The stages of cervical carcinogenesis are relatively well understood making it possible to estimate the average DPP duration (3). However, interindividual variation in the DPP duration, or misspecification of the DPP duration or its precancerous phase, are potential sources of bias in case-control studies of the effectiveness of screening for cancer prevention (4, 5).

For a sensitive screening modality, women diagnosed with cervical cancer will be far less likely than their controls to have a screening test performed during the precancerous phase of the DPP – had they had a test during this period, the precancerous lesion could have been treated and the cancer could have been prevented. A screening test during the occult invasive phase of the DPP will not prevent the cancer but is likely to lead to detection at an early stage thereby potentially reducing morbidity and mortality (6).

Valid case-control studies of screening aim to ignore non-screening tests in analyses. In practice it can be challenging to accurately determine test indication, as information may not be accurate (e.g., self-reported test indication from interviews or questionnaires) or available (e.g., limited data from administrative claims or screening databases). Knowledge of the time from test to cancer detection can help to infer whether or not it was a screening test.

There have been several previous efforts to summarize the association between screening and cervical cancer incidence. A 2005 International Agency for Research on Cancer (IARC) review of cervical cancer screening summarized the association between “ever” having been screened and the risk of cervical cancer (7). In 2013, Peirson and colleagues (8) reviewed literature published between April 1995 and April 2012 in order to assess the association between screening and risk of cervical cancer incidence and mortality. They also examined associations with varying screening intervals and the age at which screening began and ended. A third review by Meggiolaro and colleagues (9) in 2016 aimed to quantify the association for cytology screening and identify potential sources of heterogeneity. Both the Peirson and Meggiolaro reviews reported high levels of heterogeneity between included studies. Meggiolaro and colleagues stratified results by study quality, cervical cancer histology, and calendar year of screening in order to explain the observed heterogeneity. None of these previous reviews considered screening exposure definitions and differences across them.

We conducted a systematic review of case-control studies evaluating the effectiveness of screening to reduce cervical cancer incidence in order to classify the spectrum of screening exposure definitions used in these studies. Our review updates literature from the prior reviews by including publications from January 2012 to December 2018. Our goal was to better understand the implications of various screening exposure definitions on results across these case-control studies to better inform screening evaluations and audits of screening programs designed to quantify the impact of screening on cervical cancer prevention.

## Materials and Methods

This systematic review was undertaken and reported in adherence to the Preferred Reporting Items for Systematic reviews and Meta-Analyses (PRISMA) guidelines. A protocol for this systematic review was developed prior to conducting the searches (Supplementary Table S1).

In addition to considering all studies from the IARC (7), Peirson (8), and Meggiolaro (9) reviews for inclusion, we used broad search criteria similar to those used by Peirson and colleagues (8) to search PubMed Central, Ovid MEDLINE and Embase (Ovid) databases for additional studies published from January 1, 2012 to December 6, 2018. Our search included the following terms (and their derivatives): cervix uteri, cervical intraepithelial neoplasia, Papillomavirus infection, Papanicolaou (Pap/smear), screening and early detection. The present search differed from the Peirson protocol only in the exclusion of literature in languages other than English and limitation of the search from January 1, 2012 onward. In addition, reference lists from included manuscripts and from the three previous reviews were searched.

Two investigators (A. Castanon and A.W.W. Lim) independently reviewed titles and abstracts of identified articles for eligibility for full-text review. Disagreements were resolved by consensus between the two reviewers. Full-text articles of eligible abstracts were retrieved and also independently reviewed for inclusion by the same investigators. Excluded studies included those in which any study subjects were less than 15 years of age, study subjects were not offered either conventional or liquid-based cervical cytology as a screening test or did not include a comparison group who had the opportunity to be screened, but did not have cervical cancer. Study designs other than case-control and studies that examined an outcome other than cervical cancer incidence were excluded. Books, conference abstracts, narrative review articles, and articles without individual level data on screening exposure were also excluded.

Included studies were quality-appraised with the Newcastle-Ottawa Scale (10). Information to assess the quality of evidence was abstracted into a pre-specified table in duplicate (by A. Castanon and A.W.W. Lim) from the primary methodology paper for each study. The two investigators extracting these data were blind to each other's quality ratings, and rating disagreements were resolved by consensus or consultation with a third investigator (P. Sasieni).

Characteristics of included studies were abstracted into structured tables and included: lead author, publication year, country of study setting, age range of cases and controls, diagnosis years for cases, number of cases, FIGO stage and/or histological type of cases, screening organization (opportunistic or following an invitation), data sources for outcome and exposure assessment, control eligibility criteria, screening exposure measure, screening intervals studied, and measures of association.

Measures of association from included studies were most frequently reported as odds ratios (OR) for screening relative to no screening, but occasionally as relative protection (i.e., the reciprocal of the OR). Results originally reported as relative protection (RP) were converted to odds ratios by division (1/RP). When the reference group was the most frequently screened group, all ORs were divided by the OR in the least screened group. Therefore, the OR of 1.00 in the “never screened” group has an associated confidence interval (CI), and no CI is associated with the OR for the most screened group. When studies did not report ORs, the Altman method (11) was used to calculate ORs and associated standard errors and 95% CIs when sufficient information was available within the manuscripts.

The qualitative synthesis was carried out using a framework comprised of two elements: (i) description of the measures of screening exposure reported in the literature, and (ii) description of methodological differences which could have an impact on the association between screening and cervical cancer among studies reporting the same screening exposure.

## Results

**Figure 1.**
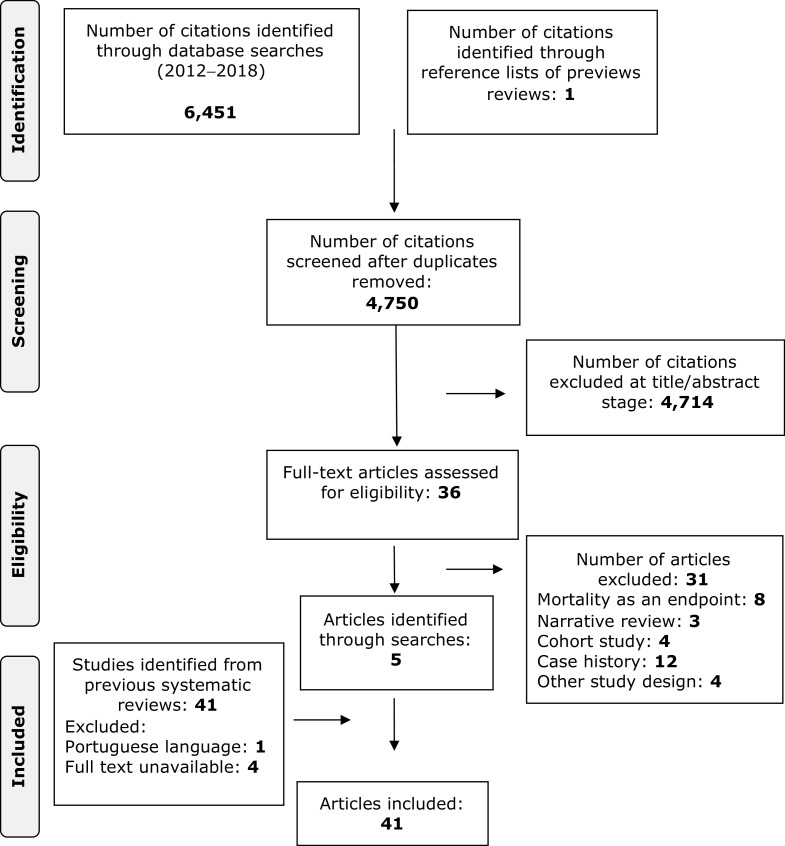
PRISMA flow diagram detailing database searches, abstracts screened, and full texts retrieved and included in the systematic review.

The database searches identified 1,384 records in PubMed, 2,553 in Ovid MEDLINE and 2,514 in Embase (Ovid) for a total of 6,451 citations (Fig. 1). In addition 41 citations were identified by searching previous systematic reviews and their reference lists. After 1,701 duplicates were removed, a total of 4,750 records remained. Title and abstract review excluded 4,714 records because they did not meet study inclusion criteria. Full text was reviewed for 36 manuscripts of which eight had a cervical mortality endpoint, three were narrative reviews, 20 were not case–control studies. In addition 41 manuscripts were identified from previous reviews, full-text was unavailable for four (12–15) and one was not in English (16). The database searches yielded a total of four manuscripts published from 2012 to 2018 that met our study inclusion criteria, 36 manuscripts from the IARC, Pierson and Meggiolaro reviews, and 1 from reference list searches. Thus, 41 manuscripts were included, likely representing 33 unique studies. Manuscripts by Sasieni and colleagues (*n* = 4; refs. 17–20) and Castanon et al. (*n* = 2; refs. 21, 22) are from the same case-control study, and the manuscripts by Celetano and colleagues (23) and Klassen and colleagues (24) appear to report on the same population, but this cannot be confirmed from the publications.

Supplementary Table S2 lists the key characteristics of included manuscripts. Studies included cases diagnosed from 1959 to 2014 from 17 different countries. Most studies included a broad age-range of women, and two studies focused exclusively on women 55 and older (22, 25). Twenty-two studies were conducted in non-invitational screening settings, seventeen studies were conducted in invitational settings, and two studies were conducted in settings with both non-invitational and invitational screening. Eight studies restricted analyses to International Federation of Gynecology and Obstetrics (FIGO) stage 1B or worse cervical cancers and five reported histology-specific results. Six studies did not specify any matching criteria, sixteen obtained screening information through interviews, four through both interviews and medical/screening records, and twenty-one through medical/screening records or databases.

Full details on the risk of bias for included studies are shown in the supplementary material (Supplementary Table S3). The risk of bias varied considerably between studies but did not seem to be influenced by the number of cervical cancers included (Supplementary Table S2).

The case-control studies with the least likelihood of bias were those in which cancers were identified through hospital pathology databases or cancer registries and in which screening histories for both cases and controls were extracted from electronic databases, screening registries, or medical records. Similarly, studies in which controls were identified through electronic databases (allowing the inclusion of all, or a sample of all, eligible women) rather than through population lists (which require the investigator to contact participants for an interview or provide a questionnaire to obtain information) were the least susceptible to bias. Six of the included studies had Newcastle-Ottawa scores of less than six and are therefore particularly susceptible to bias (24, 26–30). Eleven studies had low risk of bias (Newcastle-Ottawa scores of eight or nine) and the remaining 24 studies had a moderate risk of bias.

We identified six definitions of screening exposure among the manuscripts included in this review: “ever having a test”, “time since last negative test”, “number of tests”, “maximum interval between tests”, “screening history”, and “screening in a three year age band and risk of cancer over a five-year period”. Several manuscripts reported more than one definition of screening exposure. Since most studies attempted to exclude diagnostic tests from analyses, we use the terms “screening” and “testing” interchangeably throughout this manuscript except within the tables where the distinction is important when interpreting results.

### Ever having a test

Twenty-nine of the included studies examined the risk of cervical cancer associated with ever having a test during various look-back windows prior to cases' diagnosis date or corresponding reference date for controls (Table 1). Supplementary Figure S2 illustrates the look-back window during which screening history is ascertained when measuring ‘ever having a test’.

**Table 1.** Case–control studies reporting screening exposure as “ever having a test”.

				Look-back periods studied	
					
Author, year	Country	Age range	Measure of screening exposure/Stage	Reference/Comparison	OR (95% CI)
Andersson- Ellstrom, 2000 (57)	Sweden	20–99	Ever/never tested (no exclusions)	Not tested within 6 y/	1.00^a^
			All stages	<3 y	0.81 (0.48–1.36)
			Stratified by stage	3–6 y	0.89 (0.35–2.28)
					1.00
Andrae, 2008 (36)	Sweden	20–99	Ever/never screened (≤6 m excluded)	Not screened^b^/	0.21 (0.16–0.28)
				Screened (II+)	0.48 (0.42–0.55)
				Screened (all)	
Aristizabal, 1984 (58)	Colombia	16–60	Ever/never screened (≤12 m excluded)	Not screened within 6 y/	1.00
					0.05 (0.03–0.09)
			All stages	Screened	
Celetano^c^, 1988 (23)	USA	22–84	Ever/never screened (diagnostic tests excluded)	Never^d^/	1.00
			All stages	≤3 y	0.29 (0.15–0.58)
				Never^d^/	1.00
Chichareon, 1998 (27)	Thailand	All ages	Ever/never tested (no exclusions)	<5	0.4 (0.2–0.9)
			All stages	≥5	0.5 (0.2–0.9)
Cohen, 1993 (59)	Canada	25–64	Ever/never screened (≤12 m excluded)	Not screened within 10 y/	1.00
				Screened ≤10 y	0.43 (0.32–0.57)
			All stages		
				Not screened within 5 y/	1.00
Crocetti, 2007 (60)	Italy	25–74	Ever/never screened (≤12 m excluded)	1–3 y	0.10 (0.04–0.28)
				3–5 y	0.35 (0.16–0.77)
			All stages		
Decker, 2009 (61)	Canada	18+	Ever/never screened (≤6 m excluded)	Not screened within 5 y/	1.00
				Screened <5 y	0.36 (0.30–0.43)
			All stages		
Herrero, 1992 (26)	Latin America	<70	Ever/never screened (≤12 m excluded)	Never^d^/	1.00^a^
			All stages	Screened	0.40 (0.30–0.48)
				Not screened within 9 y/	1.00 (0.37–2.68)^e^
Hernandez-Avila, 1998 (62)			Ever/never screened (≤12 m excluded)	1–2 y	0.17 (N/A)
	Mexico	<75	All stages	3–4 y	0.35 (0.13–0.90)
				5–9 y	0.35 (0.11–1.05)
				**Never** **^d^** **/Screened**	**0.38 (0.28–0.52)**
				Never/	1.00
				<5 y	0.3 (0.2–0.4)
Hoffman, 2003 (44)	South Africa	<60	Ever/never tested (no exclusions)	5–9 y	0.3 (0.2–0.4)
			FIGO 1B+	10–14 y	0.4 (0.3–0.5)
				>15 y	0.5 (0.4–0.7)
				**Never** **^d^** **/Tested**	**0.3 (0.3–0.4)**
				Never/	1.00
Jimenez-Perez, 1997 (63)	Mexico	<70	Ever/never screened (≤12 m excluded)	1–2 y	0.2 (0.1–0.4)
			FIGO 1B+	2–5 y	0.2 (0.1–0.5)
				>5 y	0.5 (0.3–0.9)
				**Never** **^d^** **/Screened**	**0.3 (0.2–0.4)**
Kamineni, 2013 (25)	USA	55–79	Ever/never screened. Various exclusion periods presented (≤12 m, ≤6 m, ≤18 m, and ≤2 y).	Not screened within 7 y/	1.00
					
				Screened in the last 6 y (tests within 12 m excluded)	0.33 (0.12–0.92)
			All stages		
				Never^d^/	1.00
				<6 m	1.84 (0.89–3.80)
Kasinplila, 2011 (33)	Thailand	30–64	Ever/never tested (no exclusions)	6–11 m	1.53 (0.61–3.84)
			All stages	12–35 m	0.27 (0.13–0.57)
				>36 m	0.37 (0.17–0.81)
Klassen^c^, 1989 (24)	USA	45–84	Ever/never screened (≤12 m excluded)	Screened 11+ or never/	1.00
			All stages	1–2 y	0.07 (0.03–0.17)
				2–4 y	0.14 (0.05–0.39)
				5–10 y	0.38 (0.15–0.98)
				Never^d^/	1.00
La Vecchia, 1984 (45)	Italy	23–74	Ever/never tested (no exclusions)	<3 y	0.26 (0.17–0.49)
			All stages	3–5 y	0.33 (0.14–0.80)
				>5 y	0.34 (0.16–0.42)
Lonnberg, 2012 (64)	Finland	All ages	Ever/never screened (≤6 m excluded)	Not screened within 5 y/	1.00
			All stages	Screened <5 y	0.53 (0.46–0.62)
Nieminen, 1999 (29)	Finland	30–91	Screened within the population screening program	**Never/**	1.00
				**Screened**	0.38 (0.26–0.56)
			All stages		
				Never/	1.00
Olesen, 1988 (31)	Denmark	20+	Ever/never screened (≤6 m and diagnostic tests excluded)	≤3 y	0.15 (0.06–0.33)
				4–5 y	0.33 (0.07–1.50)
			All stages	>5 y	0.67 (0.23–1.86)
				**Never** **^d^** **/Screened**	**0.27 (0.18–0.42)**
				Never/	1.00
				≤3 y	0.34 (0.12–1.00)
Palli, 1990 (48)	Italy	<75	Ever/never screened (≤6 m excluded)	4–5 y	0.33 (0.09–1.27)
			All stages	>5 y	0.23 (0.09–0.58)
				**Never** **^d^** **/Screened**	**0.15 (0.09–0.25)**
Parazzini, 1990 (50)	USA	20–74	Ever/never screened (diagnostic tests excluded)	Screened 5+ y or never/	1.00 (0.71–1.40) ^e^
				<2 y	0.32 (N/A)
			All stages		
				2–5 y	0.28 (0.21–0.38)
				**Age 45–54**	**Age 45–54**
Parazzini, 1990 (49)	Italy	22–74	Ever/never screened (diagnostic tests excluded) by age group	Screened 5+ y or never/	1.00
				<3 y	0.49 (0.27–0.88)
			All stages	3–5 y	0.39 (0.16–0.97)
Sasieni^f^, 1996 (17)	UK	>20 y	Ever/never screened (≤6 m excluded)	Screened 5+y or never/	1.00
				6–11 m	0.89 (0.50–1.60)
			FIGO 1B+	1–2 y	0.53 (0.30–0.92)
				2–3 y	0.40 (0.23–0.70)
				3–4 y	0.35 (0.19–0.63)
				4–5 y	0.67 (0.38–1.19)
				**Age 40–54**	**Age 40–54**
				Never/	1.00
				0.5–1.4 y	0.38 (0.26–0.54)
				1.5–2.4 y	0.22 (0.14–0.34)
Sasieni^f^, 2003 (18)	UK	20–69	Ever/never screened (≤6 m excluded)	2.5–3.4 y	0.34 (0.23–0.50)
			FIGO 1B+	3.5–4.4 y	0.28 (0.17–0.47)
				4.5–5.4 y	0.61 (0.37–1.01)
				5.5–6.4 y	0.80 (0.40–1.60)
				≥6.5 y	1.10 (0.58–1.77)
Sasieni^f^, 2009 (19)	UK	20–69	Ever/never screened (≤6 m excluded) FIGO 1B+	Never/ ≤10 y	1.00
					0.37 (0.32–0.41)
				Not screened within 10 y/	1.00 (1.31–3.66) ^e^
Shy, 1988 (30)	USA	31–75	Ever/never screened (≤1 m excluded)	1 y	0.15 (N/A)
			Symptomatic FIGO 1B+	2 y	0.15 (0.06–0.35)
				3 y	0.36 (0.14–0.93)
				4–10 y	0.41 (0.17–0.98)
Van der Graaf, 1988 (32)	Netherlands	<70	Ever/never screened (≤12 m and diagnostic tests excluded).	Never/	1.00
				2–5 y	0.18 (0.05–0.62)
			FIGO 1B+	>5 y	0.33 (0.09–1.02)
				**Never^d^/Screened**	**0.32 (0.12–0.80)**
Wangsuphachart, 1987 (28)	Thailand	15–54	Ever/never screened (≤6 m excluded)	Never^d^/	1.00
			All stages	2–5 y	0.39 (0.21–0.74)
				Never/	1.00
Zappa, 2004 (54)	Italy	<70	Ever/never screened (≤12 m excluded)	1–3 y	0.25 (0.15–0.42)
			All stages		
				3–6 y	0.34 (0.21–0.56)
				≥6 y	0.56 (0.38–0.82)

^a^Estimated via the Altman method.

^b^The screening interval explored in this study is 0.5 to 3.5 years in women 53 or younger and 0.5 to 5.5 years in those aged 54–65; the corresponding reference group is not screened during the recommended interval.

^c^Celetano et al. and Klassen et al. appear to report on the same population, but this cannot be confirmed from the publications.

^d^Note that when interviews are used to establish screening history, it is not possible to ascertain the look-back window under study. Where screening databases were used and the period is not specified, the look-back period will be from the age at which screening is first offered to the date when the registry was created.

^e^The baseline group in these studies is the most screened group. Here the ORs have been divided by the OR in the least screened group. Note that therefore the OR of 1.00 in the “never screened” group has an associated confidence interval, and there is no CI for the most screened group.

^f^From the same case–control study.

Most studies which considered screening exposure as ‘ever having a test’ (*n* = 25) examined look-back windows less than 10 years, two studies considered 10-year look-back windows, and two studies only examined lifetime screening exposure. Most studies excluded tests within 1 month (*n* = 1), 6 months (*n* = 10), and 12 months (*n* = 9) prior to the diagnosis/reference date. Few explicitly acknowledge whether these exclusions are to eliminate symptomatic tests and/or to exclude tests taken during the occult invasive DPP. Of the studies that did not explicitly specify an exposure exclusion period, three studies excluded all self-reported diagnostic tests and six did not exclude any tests prior to diagnosis/reference date. Olesen and colleagues (31) and Van der Graff and colleagues (32) were the only studies to exclude both tests within 6 and 12 months of diagnosis/reference date, respectively, *and* any tests taken in response to symptoms during the precancerous phase of the DPP.

The impact of including symptomatic tests and those occurring during the occult invasive DPP is demonstrated by results from Kasinpila and colleagues (33) In this study, the risk of cervical cancer (although not statistically significant) was 53–84% higher among those tested between 6–11 months and within 6 months prior to diagnosis/reference date, respectively. However a 73% reduction in cervical cancer risk was seen when the test occurred 1–3 years prior to diagnosis.

Using a 7-year look-back, Kamineni and colleagues (25) was the only study to perform sensitivity analyses using various estimates of the occult invasive DPP (≤6 months, ≤12 months, 18 months and 24 months), and examined testing only during corresponding estimates of the precancerous phase of the DPP(5 yrs, 5.5 yrs, 6.0 yrs and 6.5 yrs). Their results were robust to these estimates of the occult invasive DPP.

Others use the exposure exclusion period to remove symptomatic tests from analysis and attempt to account for the effect of tests taken during the occult invasive DPP by restricting analysis to cancers International Federation of Gynaecology and Obstetrics (FIGO) stage 1B or worse (17, 18) (which are less likely to be screen-detected) or present results by FIGO stage at diagnosis (21).

In studies that reported results by various look-back windows, we noted that the reference group differed across studies. Some reference groups only included women who had never been screened or tested during the look-back window, while others also included those screened or tested prior to the look-back window.

Among the 11 studies with look-back windows of 10 or more years, the average reduction in risk associated with screening was 67% (range 57–85%). Among the 24 studies reporting results for having a test during the 2 to 6 years prior to diagnosis/reference date, the average reduction in risk associated with screening was 66% (range 11–95%).

### Time since last negative test

Fourteen studies reported results for time since last negative test (Table 2). A negative test will not have prevented cancer but will identify those at a period of lower risk than their unscreened counterparts. Studying how this risk increases over time, can inform appropriate screening intervals.

**Table 2.** Case–control studies reporting screening exposure as “time since last negative test”.

				Look-back periods studied	
Authors, year	Country	Age range	Measure of screening exposure/Stage	Reference/Comparison	OR (95% CI)
Andrae, 2008 (36)	Sweden	20–99	Negative test <6 y	Not screened/	1.00
			(≤6 m excluded)	Screened within recommended interval^a^	0.35 (0.30–0.40)
			All stages		
					**Stage 1A|Stage1B**
Castanon^b^, 2016 (21)	UK	30–69	Time since last negative	Not screened or ≥7 y/	1.00 | 1.00
			(no exclusions)	<3 y	0.02 (0.02–0.03) | 0.05 (0.04–0.06)
			(SCC results are presented here)	3–5 y	0.88 (0.77–1.01) | 0.51 (0.43–0.59)
				5–7 y	1.09 (0.91–1.30) | 0.55 (0.45–0.68)
				Never tested/	1.00
IARC Working Group, 1986 (34)	9 centers across Europe and Canada	All ages	Time since last negative	1–2 y	0.08 (0.05–0.13)^c^
			(no exclusions)	2–3 y	0.13 (0.08–0.19)
			(in women with two or more	3–4 y	0.19 (0.13–0.28)
			negatives)	4–5 y	0.36 (0.25–0.53)
			All stages	5–6 y	0.28 (0.17–0.48)
				Not screened or negative ≥10 y^d^/	1.00 (0.46–2.15)^e^
Ibanez, 2015 (39)	Spain	23–96	Time since last negative (≤6 m	≤3 y	0.38 (N/A)
			excluded)	>3 y	0.73(0.35–1.42)
			All stages	**Not screened/**	
				**Screened negative <10 y**	**0.10 (0.07–0.12)**
Kamineni, 2013 (25)	USA	55–79	Time since last negative	Not screened or ≥7 y/	1.00
			(no exclusions)	<1 y	0.09 (0.03–0.24)
			All stages	1–3 y	0.25 (0.12–0.52)
				3–5 y	0.15 (0.04–0.58)
				5–7 y	1.21 (0.37–3.90)
Macgregor, 1994 (40)	Scotland	25–60	Time since last negative	No negative ever^d^/	1.00
			(no exclusions)	≤3 y	0.30 (0.20–0.46)^e^
			All stages		
				Negative ≥5 y/	1.00
Makino, 1995 (37)	Japan	35–79	Time since last negative	1 y	0.09 (0.06–0.16)
			(excludes tests at	2 y	0.17 (0.08–0.34)
			diagnosis)	3 y	0.67 (0.26–1.73)
			All stages	4 y	0.45 (0.13–1.59)
				**Never^d^/Ever**	**0.14 (0.09–0.23)**
Miller, 2003 (65)	USA	20+		No negative/	1.00
				0–18 m	0.12 (0.09–0.18)^f^
			Time since last negative	19–30 m	0.22 (0.14–0.35)
			(no exclusions)	31–42 m	0.25 (0.14–0.43)
			All stages	3.6–4.9 y	0.35 (0.21–0.58)
				5–10 y	0.53 (0.34–0.84)
				>10yrs	0.86 (0.52–1.44)
Mitchell, 2003 (41)	Australia	<70	Time since last negative	Negative ≥3 y^d^/	1.00
			(no exclusions)	<3 y	0.33 (0.20–0.54)^f^
			All stages		
				Not screened or negative ≥5.5 y/	1.00
Sasieni^b^, 1996 (17)	UK	>20	Time since last negative	1 y	0.33 (0.18–0.61)
			(no exclusions)	2 y	0.26 (0.14–0.47)
			FIGO 1B+ only	3 y	0.32 (0.17–0.56)
				4–5.4 y	0.64 (0.36–1.14)
Sasieni^a^, 2003 (18)	UK	20–69	Time since last negative		**20–39|55–69**
			(no exclusions)	Never or no negative/	1.00 | 1.00
			FIGO 1B+ only	0–2.9 y	0.28 (0.20–0.41) | 0.13 (0.08–0.19)
				3.0–4.9 y	1.03 (0.68–1.56) | 0.20 (0.12–0.33)
				≥5 y	2.05 (1.20–3.49) | 0.45 (0.25–0.81)
Sasieni^a^, 2009 (19)	UK	20–69	Time since last negative	Never or >7.5 y/	1.00
			(no exclusions)	2.5–3.4 y	0.15 (0.12–0.19)
			(SCC results are presented here)	3.5–4.4 y	0.27 (0.21–0.35)
				4.5–5.4 y	0.33 (0.24–0.44)
			FIGO 1B+ only	5.5–7.5 y	0.55 (0.42–0.73)
Sobue, 1988 (38)	Japan	30–79	Negative test <10 years	No negative^d^/	1.00
			(no exclusions)	<10 years	combined 0.41(0.13–1.29)
			All stages		symptomatic 0.41(0.11–1.56)
					screened detected 0.43(0.05–3.71)
Zhang, 1989 (43)	China	40–79	Time since last negative test	Tested negative ≥6 y^d^/	1.00
			(exclusions unclear)	<2 y	0.16 (0.05–0.58)
			FIGO 1B+ only		

^a^The screening interval explored in this study is 0.5 to 3.5 years in women 53 or younger and 0.5 to 5.5 years in those aged 54-65.

^b^Manuscripts by Castanon et al. and Sasieni et al. (*n* = 5) are based on the same case-control study, which is an ongoing audit.

^c^1/relative risk.

^d^These studies include only screened women.

^e^The baseline group in these studies is the most screened group. Here the ORs have been divided by the OR in the least screened group. Note that therefore the OR of 1.00 in the “never screened” group has an associated confidence interval, and there is no CI for the most screened group.

^f^Estimated via the Altman method.

There are a number of methodological issues that can introduce bias in this measure of screening exposure. Only negative tests after which no further action is recommended should be considered for this analysis. The manuscripts by IARC (34), Kamineni and colleagues (35), Andrae and colleagues (36), and all manuscripts by Sasieni and colleagues and Castanon and colleagues, included only negative tests that did not lead to further action. We note that the IARC study (34) is a pooled analysis of case-control studies with similar designs and a common measure of screening exposure.

Another methodological difference is the composition of the reference group. Some studies included those with no negative tests (*n* = 4) in the reference group, others only included women with no tests (*n* = 2), and others include women with tests outside the intervals under study (*n* = 3). Five other studies included both those with no tests and those with tests outside the intervals under study. The different composition of the reference groups can partly be attributed to the fact that the studies by Makino (37), Sobue (38), Ibanez (39), Macgregor (40), Mitchell (41), Yang (42) and Zhang (43) included screened women only.

Using the shortest time interval between testing and cancer diagnosis for each study, the average reduction in risk associated with a negative screen was 77% (range 59–95%). However, the observed reduction in risk decreased with increasing time between diagnosis/reference date in all studies.

### Number of tests

Eight studies reported results by number of tests (Table 3). Five of these eight studies were agnostic to test results while three restricted analyses to negative tests. We note that, as in the previous exposure definition, the composition of the reference group differs between studies. Tests during the occult invasive DPP are not excluded in this definition of screening exposure because it is not attempting to make reference to the time between the test and diagnosis/reference date. Instead, this screening exposure definition evaluates whether the risk is the same after receipt of the first primary screening tests as it is after a series of negative primary screening tests (i.e., a dose response). Triage and surveillance tests should be excluded.

**Table 3.** Case–control studies reporting screening exposure as “number of tests”.

				Number of previous tests	
Authors, year	Country	Age range	Measure of screening exposure^a^/Stage	Reference/Comparison	OR (95% CI)
Hoffman,	South Africa	<60	Number of previous	No tests/	1.00
2003 (44)			tests	1	0.4 (0.3–0.6)
			(no exclusion)	2	0.2 (0.1–0.3)
			FIGO 1B+	≥3	0.2 (0.2–0.3)
IARC Working Group, 1986 (34)	9 centers across Europe and Canada	All ages	Number of negative tests	No negative/	1.00
				1	0.20 (N/A)
			(no exclusions)	≥2	0.09 (N/A)
			FIGO 1B+		
La Vecchia, 1984 (45)	Italy	23–74	Number of previous	No tests/	1.00
			tests	1	0.56 (0.29–1.08)
			(no exclusions)	≥2	0.19 (0.10–0.35)
			All stages		
Macgregor, 1994 (40)	Scotland	25–60	Number of negative	No negative/	1.00
			tests	1–3	0.35 (0.25–0.49)^b^
			(no exclusions)	4–6	0.19 (0.12–0.31)
			All stages		
Mitchell, 2003 (41)	Australia	<70	Number of negative	One negative/	1.00
			tests	2	0.99 (0.50–1.96)^b^
			(no exclusions)	3	0.66 (0.33–1.32)
			All stages	4	0.50 (0.22–1.15)
				5	0.46 (0.20–1.08)
				≥6	0.56 (0.31–1.00)
Palli, 1990 (48)	Italy	<75	Number of previous	No screens/	1.00
			tests	1	0.29 (0.15–0.55)
			(≤6 m excluded)	2	0.13 (0.05–0.31)
			All stages	≥3	0.06 (0.03–0.16)
Parazzini, 1990 (50)	USA	20–74	Number of previous screens <10 y (diagnostic tests excluded)	No screens <10 y/	1.00
				1–2	0.40 (0.26–0.61)
				≥3	0.29 (0.15–0.33)
			All stages		
Parazzini, 1990 (49)	Italy	45–54 (shown here)	Number of previous screens by age group	No screens/	1.00
				1–2	0.50 (0.25–1.00)
			(diagnostic tests	≥3	0.24 (0.12–0.47)
			excluded) All stages		

^a^Note that all studies in this table except for McGregor and Mitchell used interviews to establish screening history. When using interviews, it is not possible to ascertain the look-back window under study. Where screening databases were used and the period is not specified, the look-back period will be to the age at which screening is first offered from the date when the registry was created.

^b^Estimated via the Altman method.

All studies with this screening exposure definition observed decreasing risk of cervical cancer with increasing number of tests prior to diagnosis/reference date. The average reduction in risk associated with two or more tests was 79% (range 54–94%).

### Other screening exposure definitions

The most common other definition of screening exposure was that which established how often women attended screening (Table 4). This definition is useful to assess how testing intensity changes the risk of cervical cancer. Four studies reported on the frequency at which women were screened (21, 22, 28, 42). For example, Yang and colleagues (42), classified women depending on whether they only attended one of the last two screening rounds (i.e., not regularly screened) or whether they attended both of the last two screening rounds. This classification was done irrespective of test result. In contrast to ‘number of tests’ this measure does take into account the intervals at which tests were taken, hence all four studies excluded tests taken during the estimated occult invasive DPP before establishing how often women had attended screening. More frequent screening was associated with a lower risk of cervical cancer compared to irregular attendance.

**Table 4.** Case–control studies reporting other measures of screening exposure.

				Look-back periods studied	
Authors, year	Country	Age ranges included	Measure of screening exposure/Stage	Reference/Comparison	OR (95% CI)
Castanon^a^, 2014 (22)	UK	65–83	Maximum interval between tests taken at ages 50–	Not screened <1 y/	1.00
			64 y	<3.5 y	0.27 (0.21–0.34)
			All stages	3.5–5.4 y	0.25 (0.20–0.30)
				5.5–8.9 y	0.34 (0.26–0.43)
				9.0–15 y	0.54 (0.40–0.71)
Castanon^a^, 2014 (22)	UK	65–83	Screening history from age 50–64 years	Not screened aged 50–64/	1.00
			All stages	Adequate negative	0.16 (0.13–0.19)
				Inadequate but negative	0.34 (0.28–0.42)
				Abnormal	1.83 (1.37–2.43)
Castanon^a^, 2016 (21)	UK	30–69	Screening history	Not screened/	1.00
			(≤6 m excluded)	Screened up to date	0.05 (0.04–0.06)
			All stages	(negative in the last 3.5 or 5.5 y)	0.40 (0.33–0.47)
			(SCC, stage II+ results presented here)	Screened lapsed	
				(not within 3.5 or 5.5 y or abnormal without timely follow-up)	
Sasieni^a^, 2009 (19)	UK	20–69	Maximum interval between tests	Not screened or >5.5 y/	1.00
			All stages	<3.5 y	0.25 (0.21–0.29)
			(SCC results are presented here)	3.5–5.5	0.39 (0.33–0.45)
Sasieni^a^, 2009 (20)	UK	20–69	Screened in a 3-year period (age 32–34 y) vs. not screened in a 5-year period (age 30–34 y) and risk of cervical cancer in the following five years (age 35–39 y)	Not screened 30–34^b^/	1.00
				Screened age 32–34	0.55 (0.44–0.69)
Yang B, 2008 (42)	Australia	20–69	Test history	Not screened <4 y/	1.00
			(≤3 m excluded)	Regular (both bienniums)	0.04 (0.03–0.06)
			All stages	Irregular (one biennium only)	0.15 (0.12–0.18)
Wangsuphachart, 1987 (28)	Thailand	15–54	Test history	Not screened/	1.00
			(≤6 m excluded)	1 test only	0.92 (0.58–6.46)
			All stages	A screen every 2–5 y	0.39 (0.21–0.74)
				A screen every year	0.25(0.12–0.59)

Abbreviation: SCC, squamous cell carcinoma.

^a^Manuscripts by Castanon at al. and Sasieni et al. are based on the same case–control dataset, but examine different age groups.

^b^This study looked at equivalent comparisons across overlapping age ranges from 20 to 69 years.

A problem with screening exposure definitions looking at the time since last test is that women on annual or six-monthly follow-up or repeat schedules will have a short time since last test. Such simple classification is likely to falsely make screening appear less effective (since such women are at increased risk of cervical cancer).

Defining exposure as the “maximum interval between tests” (Supplementary Fig. S3) can address the issue raised above. For example, consider a look-back window of 6 years and a woman who had three tests six months apart, but whose previous test was five years prior to these; her maximum interval would be five years. No attention is given to her most recent interval (6 months) or her average interval (2 years). Had this woman's only test been the one 6 months prior to diagnosis, her interval would have been 5.5 years and she would have been grouped with those who had no tests. In this review two studies used this measure of screening exposure (19, 22).

All previous screening exposure definitions have defined the age-groups by the age of the cases and controls (i.e., age at diagnosis/reference date) rather than the age at screening. In order to specifically examine the benefit of screening within a specific age range (e.g., ages 20–24), a different approach is required. The final screening exposure definition identified in this review considered whether a woman had been screened in a narrow three-yearly age band (e.g., 40–42 years vs. not screened 40–44 years) and then looked at whether she developed cancer in the subsequent five years (e.g., 45–49 years; Supplementary Fig. S4). To date, this screening exposure definition has only been studied by Sasieni and colleagues (20).

### Studies with more than one screening exposure definition

Studies using more than one screening exposure definition illustrate the differences in magnitude of observed associations based on the choice of exposure definition. Hoffman and colleagues (44) reported a 70% lower risk of cervical cancer among women ever tested but an 80% lower risk among those who had three or more tests.

Andrae and colleagues (36) reported a 52% lower risk among those tested as recommended compared with a 65% lower risk among those who tested negative. Sasieni and colleagues (18) observed an even greater difference: 66% lower risk 2.5–3.4 years after a test, but an 87% lower risk within 3 years of a negative test.

Manuscripts by IARC (34), La Vecchia and colleagues (45), Macgregor and colleagues (46), Mitchell and colleagues (47), Palli and colleagues (48) and Parazzini and colleagues (49, 50) report results by time since last negative and by number of tests. All studies except for Macgregor and colleagues observed a lower risk of cervical cancer after multiple tests than for time since last negative test. For example, Palli and colleagues reported a 66% reduction in risk of cervical cancer within 3 years of a negative test, but a 94% reduction following three or more tests.

## Discussion

Our review examined data from studies in 17 countries spanning 6 continents covering cervical cancer cases diagnosed from 1959 to 2014. They varied by how often screening was offered and under what conditions (invitational or not). Choice of screening exposure definition impacted the magnitude of observed screening benefit. Cervical cancer risk on average decreased by 66% when screening exposure was defined as ever tested, by 77% when exposure was defined as time since last negative test and by 79% after two or more previous tests. Within study differences between exposure definitions estimates were even greater than between studies differences. Methodological differences that affect estimates of screening benefit within exposure definition include the estimated duration of the occult invasive DPP and the choice of reference group.

A bridging search in PubMed covering January 2019 to April 2020 identified two additional manuscripts that met the inclusion criteria for this review (51, 52). No new measures of screening exposure were reported, and results were in keeping with those presented in this review.

We compared results from different screening exposure definitions to illustrate their impact on the magnitude of the effect. Observed differences are largely due to differences in the underlying risk of cervical cancer among women included in each exposure definition. Some exposure definitions are evaluating the tests' ability to predict risk (i.e., after a negative test) and some the ability to reduce risk (i.e., participating in screening). In practice, risks from different exposure definitions should not be compared.

The population-level benefit of cervical cancer screening will depend on multiple factors including screening coverage, accuracy of the screening test, and quality of the follow-up for those testing positive (7, 53), leading prior studies to conclude that these factors may be driving observed differences in study results (7, 9). None of the previous reviews addressed screening exposure definition as a source of heterogeneity between studies. Although Peirson and colleagues (8) reported the screening exposure definition for each study, they did not stratify results by them.

The case-control design ensures that differences in screening coverage do not impact results. Defining exposure to screening as ever having a test provides the most straightforward estimate of benefit of at least one test when more specific screening history and/or test results are not available. However, study results will, to some extent, reflect the quality of the follow-up for positive tests.

Analyses focusing on negative tests will reflect the sensitivity of the screening test, allowing for estimation of appropriate screening intervals. Although knowledge of test result is needed when focusing on negative tests, assuming the test has a reasonable negative predictive value there is no need to exclude symptomatic or diagnostic tests for this exposure definition.

Other screening exposure definitions are less practical as they require data from more than one round of screening which may not always be available. However, these screening exposure measures may be more desirable for mature screening programs because they can consider multiple primary testing prior to the diagnosis/reference date and allow estimation of the benefit of repeat testing. Measures such as number of tests (in particular negative tests) and regularity of testing can be useful when comparing results from different settings. Accounting for number of past tests aims to equalize the risk among individuals and it is also a way to standardize the difference in the testing accuracy between studies. The likelihood that a third test is a false-negative is much lower than that for a single negative test (assuming sensitivity is independent between tests). Note that the advantage of two average-quality cytology tests over one could be significant, whereas the advantage of two human papillomavirus (HPV) primary tests over one may be small.

Most studies in this review excluded tests during a short period prior to diagnosis. This exclusion period was typically 6 or 12 months but varied from 1 to 24 months. Some studies indicated that the rationale was to exclude tests taken in response to symptoms, but few explicitly stated whether they (also) intended to exclude tests taken during the occult invasive DPP. The risk of cervical cancer associated with tests during the occult invasive DPP reflects the prevalence of screen-detected cancer. Determining the precise duration of the occult invasive phase is challenging and it will, of course, not be identical for every individual. In this review, only Kamineni and colleagues (25) actively focused on screening that occurred during the presumed precancerous period of the DPP. To isolate the precancerous phase, they estimated the duration of the occult invasive phase. As, the duration of the occult invasive phase will be half as long, on average, in screen-detected cases than among cases whose cancer was detected as a result of symptoms, Kamineni and colleagues (25) analysed their data in two strata: 1) symptomatic cases and controls with no screening during the presumed occult invasive phase, and 2) screen-detected cases and recently screened controls. They performed sensitivity analyses using various estimates of the occult invasive DPP, and results were robust to estimates of the occult invasive DPP of up to 2 years prior to diagnosis/reference date.

In contrast, Wang and colleagues (51) knew the majority of cervical cancer cases in their study had an abnormal test result within 6 months of diagnosis and a review of medical records found that tests within one month of diagnosis were likely to be performed because of symptoms. However instead of analysing data in the manner of Kamineni and colleagues, they excluded, for both cases and controls, tests within 6 months of diagnosis and extended the exposure window by half a year so analysis would reflect screening in the round prior to diagnosis. If screening has been only recently introduced in a population, if data for only one screening round is available, or if screening is not invitational (i.e., there is no ‘round prior to diagnosis’), excluding tests taken during the occult invasive DPP will lead to a high proportion of cases being classed as ‘never screened’. This may bias in favour of screening unless it is accounted for during the analysis by, for instance, using the analytic approach taken Kamineni and colleagues (25).

The choice of reference group is a methodological consideration that has not been given attention in the literature. The observed screening benefit will be greater if the reference group includes only women who have never been screened as opposed to a reference group that also includes women whose last test was prior to the defined look-back window.

This review did not focus on evaluation of screening effectiveness by age or by histological type. There is evidence from Italy (54), the UK (18), and South Africa (44) that screening is less effective in young women, but this has not been consistently observed (36). There is also evidence that screening with cytology (but not HPV tests) is less sensitive for detecting adenocarcinoma of the cervix (21, 51). It is likely that age and histological type will also be sources of heterogeneity when comparing results from different studies.

Many cervical cancer screening programs are transitioning from cytology-based screening to primary HPV screening. Routine evaluations of the effectiveness of primary HPV screening in preventing cervical cancer will be critical to ensure that benefits observed in randomized trials are borne out in practice. Additionally, larger cohorts of HPV-vaccinated women are becoming screen-eligible and the lower prevalence of cervical disease has already been shown to decrease the positive predictive value of screening (55).

As evidenced by the number of case-control studies in this review, this study design is largely accepted as an efficient way to quantify the benefits of cervical screening in a variety of settings. The exposure definitions identified in this review will be relevant irrespective of whether a screening program employs cytology alone, co-testing, primary HPV screening, or is transitioning to a new screening modality.

To ensure programs evaluate their progress towards cervical cancer elimination and can accurately measure the impact of transitioning to new screening modalities, case-control studies should be implemented alongside routine quality assurance measures to allow for routine evaluation of screening.

The next step is to establish international consensus for core screening exposure definitions to be used in case-control studies of screening effectiveness, similar to those established for effectiveness trials (56). This will enable the development of guidelines to standardize definitions and establish key scientific questions to be addressed under each exposure definition. Only then will consistent evaluation of cervical cancer screening programs and international comparisons be possible given the evolving cervical cancer prevention landscape.

## Authors’ Disclosures

A.W.W. Lim reports non-financial support from Copan Italia S.p.A and non-financial support from Roche Diagnostics outside the submitted work. P. Sasieni reports grants from Cancer Research UK during the conduct of the study as well as grants from Public Health England and NIHR and personal fees from Roche outside the submitted work. No disclosures were reported by the other authors.

## Disclaimer

The content is solely the responsibility of the authors and does not necessarily represent the official views of the NIH.

## References

[1] Cole P , Morrison AS. Basic issues in population screening for cancer. J Natl Cancer Inst 1980;64:1263–72. 6767876

[2] Weiss NS . Case-control studies of the efficacy of screening tests designed to prevent the incidence of cancer. Am J Epidemiol 1999;149:1–4. 9883787 10.1093/oxfordjournals.aje.a009721

[3] Schiffman M , Wentzensen N. Human papillomavirus infection and the multistage carcinogenesis of cervical cancer. Cancer Epidemiol Biomarkers Prev 2013;22:553–60. 23549399 10.1158/1055-9965.EPI-12-1406 PMC3711590

[4] Weiss NS , McKnight B, Stevens NG. Approaches to the analysis of case-control studies of the efficacy of screening for cancer. Am J Epidemiol 1992;135:817–23. 1595681 10.1093/oxfordjournals.aje.a116368

[5] Etzioni RD , Weiss NS. Analysis of case-control studies of screening: impact of misspecifying the duration of detectable preclinical pathologic changes. Am J Epidemiol 1998;148:292–7. 9690367 10.1093/oxfordjournals.aje.a009638

[6] Castanon A , Tataru D, Sasieni P. Survival from cervical cancer diagnosed aged 20–29 years by age at first invitation to screening in England: population-based study. Cancers 2020;12:2079. 10.3390/cancers12082079 PMC7463626 32731340

[7] IARC Working Group on the Evaluation of Cancer Preventative Strategies. Cervix cancer screening. IARC Handbooks of Cancer Prevention, ed. IARC Handbooks of Cancer Prevention. Vol. Volume 10. 2005, Lyon, France: IARC Press.302.

[8] Peirson L , Fitzpatrick-Lewis D, Ciliska D, Warren R. Screening for cervical cancer: a systematic review and meta-analysis. Syst Rev 2013;2:35. 23706117 10.1186/2046-4053-2-35 PMC3681632

[9] Meggiolaro A , Unim B, Semyonov L, Miccoli S, Maffongelli E, La Torre G. The role of Pap test screening against cervical cancer: a systematic review and meta-analysis. Clin Ter 2016;167:124–39. 27598026 10.7417/CT.2016.1942

[10] Wells G , Shea B, O'Connell D, Peterson J, Welch V, Losos M, . The Newcastle-Ottawa Scale (NOS) for assessing the quality of nonrandomised studies in meta-analyses. 2019; Available from: http://www.ohri.ca/programs/clinical_epidemiology/oxford.asp.

[11] Bland JM , Altman DG. Statistics notes. The odds ratio. BMJ 2000;320:1468. 10827061 10.1136/bmj.320.7247.1468 PMC1127651

[12] Sato S , Makino H, Yajima A, Fukao A. Cervical cancer screening in Japan. A case-control study. Acta Cytol 1997;41:1103–6. 9250306 10.1159/000332795

[13] Berrino F , Gatta G, d'Alto M, Crosignani P, Riboli E. Efficacy of screening in preventing invasive cervical cancer: a case-control study in Milan, Italy. IARC Sci Publ 1986;76:111–23. 3570398

[14] Clarke EA , Hilditch S, Anderson TW. Optimal frequency of screening for cervical cancer: a Toronto case-control study. IARC Sci Publ 1986;76:125–31. 3570399

[15] Talbott E , Norman S, Kuller L. Refining preventive strategies for invasive cervical cancer: a population-baased case-control study. J Women Health 2009;4:387–95.

[16] Nascimento MI , Silva GA, Monteiro GT. [Previous history of Pap smears and cervical cancer: a case-control study in the Baixada Fluminense, Rio de Janeiro State, Brazil]. Cad Saude Publica 2012;28:1841–53. 23090165 10.1590/s0102-311x2012001000004

[17] Sasieni PD , Cuzick J, Lynch-Farmery E. Estimating the efficacy of screening by auditing smear histories of women with and without cervical cancer. The National Co-ordinating Network for Cervical Screening Working Group. Br J Cancer 1996;73:1001–5. 8611418 10.1038/bjc.1996.196 PMC2075813

[18] Sasieni P , Adams J, Cuzick J. Benefit of cervical screening at different ages: evidence from the UK audit of screening histories. Br J Cancer 2003;89:88–93. 12838306 10.1038/sj.bjc.6600974 PMC2394236

[19] Sasieni P , Castanon A, Cuzick J. Screening and adenocarcinoma of the cervix. Int J Cancer 2009;125:525–9. 19449379 10.1002/ijc.24410

[20] Sasieni P , Castanon A, Cuzick J. Effectiveness of cervical screening with age: population based case-control study of prospectively recorded data. BMJ 2009;339:b2968. 19638651 10.1136/bmj.b2968 PMC2718082

[21] Castanon A , Landy R, Sasieni PD. Is cervical screening preventing adenocarcinoma and adenosquamous carcinoma of the cervix? Int J Cancer 2016;139:1040–5. 27096255 10.1002/ijc.30152 PMC4915496

[22] Castanon A , Landy R, Cuzick J, Sasieni P. Cervical screening at age 50–64 years and the risk of cervical cancer at age 65 years and older: population-based case control study. PLoS Med 2014;11:e1001585. 24453946 10.1371/journal.pmed.1001585 PMC3891624

[23] Celentano DD , Klassen AC, Weisman CS, Rosenshein NB. Cervical cancer screening practices among older women: results from the Maryland cervical cancer case-control study. J Clin Epidemiol 1988;41:531–41. 3385455 10.1016/0895-4356(88)90057-1

[24] Klassen AC , Celentano DD, Brookmeyer R. Variation in the duration of protection given by screening using the Pap test for cervical cancer. J Clin Epidemiol 1989;42:1003–11. 2809650 10.1016/0895-4356(89)90166-2

[25] Kamineni A , Weinmann S, Shy KK, Glass AG, Weiss NS. Efficacy of screening in preventing cervical cancer among older women. Cancer Causes Control 2013;24:1653–60. 23744043 10.1007/s10552-013-0239-4

[26] Herrero R , Brinton LA, Reeves WC, Brenes MM, de Britton RC, Gaitan E, . Screening for cervical cancer in Latin America: a case-control study. Int J Epidemiol 1992;21:1050–6. 1336485 10.1093/ije/21.6.1050

[27] Chichareon S , Herrero R, Munoz N, Bosch FX, Jacobs MV, Deacon J, . Risk factors for cervical cancer in Thailand: a case-control study. J Natl Cancer Inst 1998;90:50–7. 9428783 10.1093/jnci/90.1.50

[28] Wangsuphachart V , Thomas DB, Koetsawang A, Riotton G. Risk factors for invasive cervical cancer and reduction of risk by ‘Pap’ smears in Thai women. Int J Epidemiol 1987;16:362–6. 3667032 10.1093/ije/16.3.362

[29] Nieminen P , Kallio M, Anttila A, Hakama M. Organised vs. spontaneous Pap-smear screening for cervical cancer: a case-control study. Int J Cancer 1999;83:55–8. 10449608 10.1002/(sici)1097-0215(19990924)83:1<55::aid-ijc11>3.0.co;2-u

[30] Shy KK , Chu J, Mandelson M, Greer B, Figge D. Papanicolaou smear screening interval and risk of cervical cancer. Obstet Gynecol 1989;74:838–43. 2586947

[31] Olesen F . A case-control study of cervical cytology before diagnosis of cervical cancer in Denmark. Int J Epidemiol 1988;17:501–8. 3209326 10.1093/ije/17.3.501

[32] van der Graaf Y , Zielhuis GA, Peer PG, Vooijs PG. The effectiveness of cervical screening: a population-based case-control study. J Clin Epidemiol 1988;41:21–6. 3335869 10.1016/0895-4356(88)90005-4

[33] Kasinpila C , Promthet S, Vatanasapt P, Sasieni P, Parkin DM. Evaluation of the nationwide cervical screening programme in Thailand: a case-control study. J Med Screen 2011;18:147–53. 22045824 10.1258/jms.2011.011075

[34] Screening for squamous cervical cancer: duration of low risk after negative results of cervical cytology and its implication for screening policies. IARC Working Group on evaluation of cervical cancer screening programmes. Br Med J 1986;293:659–64. 3092971 10.1136/bmj.293.6548.659 PMC1341512

[35] Hakama M , Rasanen-Virtanen U. Effect of a mass screening program on the risk of cervical cancer. Am J Epidemiol 1976;103:512–7. 1274953 10.1093/oxfordjournals.aje.a112253

[36] Andrae B , Kemetli L, Sparen P, Silfverdal L, Strander B, Ryd W, . Screening-preventable cervical cancer risks: evidence from a nationwide audit in Sweden. J Natl Cancer Inst 2008;100:622–9. 18445828 10.1093/jnci/djn099

[37] Makino H , Sato S, Yajima A, Komatsu S, Fukao A. Evaluation of the effectiveness of cervical cancer screening: a case-control study in Miyagi, Japan. Tohoku J Exp Med 1995;175:171–8. 7792786 10.1620/tjem.175.171

[38] Sobue T . A case-control study of the effectiveness of cervical cancer screening in Osaka, Japan. Jpn J Cancer Res 1988;79:1269–75. 3148596 10.1111/j.1349-7006.1988.tb01555.x PMC5917660

[39] Ibanez R , Alejo M, Combalia N, Tarroch X, Autonell J, Codine L, . Underscreened women remain overrepresented in the pool of cervical cancer cases in spain: a need to rethink the screening interventions. Biomed Res Int 2015;2015:9. 10.1155/2015/605375 PMC4477117 26180804

[40] Macgregor JE , Campbell MK, Mann EM, Swanson KY. Screening for cervical intraepithelial neoplasia in north east Scotland shows fall in incidence and mortality from invasive cancer with concomitant rise in preinvasive disease. BMJ 1994;308:1407–11. 8019250 10.1136/bmj.308.6941.1407 PMC2540391

[41] Mitchell H , Hocking J, Saville M. Improvement in protection against adenocarcinoma of the cervix resulting from participation in cervical screening. Cancer 2003;99:336–41. 14681940 10.1002/cncr.11835

[42] Yang B , Morrell S, Zuo Y, Roder D, Tracey E, Jelfs P. A case-control study of the protective benefit of cervical screening against invasive cervical cancer in NSW women. Cancer Causes Control 2008;19:569–76. 18286380 10.1007/s10552-008-9118-9

[43] Zhang ZF , Parkin DM, Yu SZ, Esteve J, Yang XZ, Day NE. Cervical screening attendance and its effectiveness in a rural population in China. Cancer Detect Prev 1989;13:337–42. 2743356

[44] Hoffman M , Cooper D, Carrara H, Rosenberg L, Kelly J, Stander I, . Limited Pap screening associated with reduced risk of cervical cancer in South Africa. Int J Epidemiol 2003;32:573–7. 12913031 10.1093/ije/dyg081

[45] La Vecchia C , Franceschi S, Decarli A, Fasoli M, Gentile A, Tognoni G. Pap" smear and the risk of cervical neoplasia: quantitative estimates from a case-control study. Lancet 1984;2:779–82. 6148523 10.1016/s0140-6736(84)90705-0

[46] Macgregor JE , Moss SM, Parkin DM, Day NE. A case-control study of cervical cancer screening in north east Scotland. Br Med J 1985;290:1543–6. 3924157 10.1136/bmj.290.6481.1543 PMC1415709

[47] Mitchell HS , Giles GG. Cancer diagnosis after a report of negative cervical cytology. Med J Aust 1996;164:270–3. 8628160 10.5694/j.1326-5377.1996.tb94186.x

[48] Palli D , Carli S, Cecchini S, Venturini A, Piazzesi G, Buiatti E. A centralised cytology screening programme for cervical cancer in Florence. J Epidemiol Community Health 1990;44:47–51. 2348148 10.1136/jech.44.1.47 PMC1060596

[49] Parazzini F , Hildesheim A, Ferraroni M, La Vecchia C, Brinton LA. Relative and attributable risk for cervical cancer: a comparative study in the United States and Italy. Int J Epidemiol 1990;19:539–45. 2135870 10.1093/ije/19.3.539

[50] Parazzini F , Negri E, La Vecchia C, Bocciolone L. Screening practices and invasive cervical cancer risk in different age strata. Gynecol Oncol 1990;38:76–80. 2354830 10.1016/0090-8258(90)90015-d

[51] Wang J , Elfstrom KM, Andrae B, Nordqvist Kleppe S, Ploner A, Lei J, . Cervical cancer case-control audit: Results from routine evaluation of a nationwide cervical screening program. Int J Cancer 2020;146:1230–40. 31107987 10.1002/ijc.32416 PMC7003887

[52] Landy R , Sasieni PD, Mathews C, Wiggins CL, Robertson M, McDonald YJ, . Impact of screening on cervical cancer incidence: a population-based case-control study in the United States. Int J Cancer 2020;147:887–96. 31837006 10.1002/ijc.32826 PMC7282928

[53] Cuzick J , Clavel C, Petry KU, Meijer CJ, Hoyer H, Ratnam S, . Overview of the European and North American studies on HPV testing in primary cervical cancer screening. Int J Cancer 2006;119:1095–101. 16586444 10.1002/ijc.21955

[54] Zappa M , Visioli CB, Ciatto S, Iossa A, Paci E, Sasieni P. Lower protection of cytological screening for adenocarcinomas and shorter protection for younger women: the results of a case-control study in Florence. Br J Cancer 2004;90:1784–6. 15150597 10.1038/sj.bjc.6601754 PMC2409750

[55] Lei J , Ploner A, Lehtinen M, Sparen P, Dillner J, Elfstrom KM. Impact of HPV vaccination on cervical screening performance: a population-based cohort study. Br J Cancer 2020;123:155–60. 32362659 10.1038/s41416-020-0850-6 PMC7341799

[56] Comet Initiative. Core outcomes measures in effectiveness trials. 3 December 2020; Available from: https://www.comet-initiative.org/.

[57] Andersson-Ellstrom A . The pap-smear history of women with invasive cervical squamous carcinoma. A case-control study from Sweden. Acta Obstet Gynecol Scand 2000;79:221–6. 10716304

[58] Aristizabal N . The impact of vaginal cytology on cervical cancer risks in Cali, Colombia. Int J Cancer 1984;34:5–9. 6746118 10.1002/ijc.2910340103

[59] Cohen MM . Using administrative data for case-control studies: the case of the Papanicolaou smear. Ann Epidemiol 1993;3:93–8. 8287163 10.1016/1047-2797(93)90015-v

[60] Crocetti E , Battisti L, Betta A, Palma PD, Paci E, Piffer S, . The cytological screening turned out effective also for adenocarcinoma: a population-based case-control study in Trento, Italy. Eur J Cancer Prev 2007;16:564–7. 18090131 10.1097/CEJ.0b013e3280145c14

[61] Decker K , Demers A, Chateau D, Musto G, Nugent Z, Lotocki R, . Papanicolaou test utilization and frequency of screening opportunities among women diagnosed with cervical cancer. Open Med 2009;3:e140–7. 21603052 PMC3090124

[62] Hernandez-Avila M , Lazcano-Ponce EC, de Ruiz PA, Romieu I. Evaluation of the cervical cancer screening programme in Mexico: a population-based case-control study. Int J Epidemiol 1998;27:370–6. 9698122 10.1093/ije/27.3.370

[63] Jiménez-P rez M , Thomas DB. Has the use of pap smears reduced the risk of invasive cervical cancer in Guadalajara, Mexico? Int J Cancer 1999;82:804–9. 10446445 10.1002/(sici)1097-0215(19990909)82:6<804::aid-ijc6>3.0.co;2-n

[64] Lonnberg S , Anttila A, Luostarinen T, Nieminen P. Age-specific effectiveness of the Finnish cervical cancer screening programme. Cancer Epidemiol Biomarkers Prev 2012;21:1354–61. 22665576 10.1158/1055-9965.EPI-12-0162

[65] Miller MG , Sung HY, Sawaya GF, Kearney KA, Kinney W, Hiatt RA. Screening interval and risk of invasive squamous cell cervical cancer. Obstet Gynecol 2003;101:29–37.

